# Prevalence and risk factors for carriage of carbapenem-resistant *Acinetobacter baumannii* in post-acute care hospitals, Israel, 2021

**DOI:** 10.2807/1560-7917.ES.2025.30.15.2400563

**Published:** 2025-04-17

**Authors:** Vered Schechner, Samira Masarwa, Gabrielle D Levi, Adi Cohen, Fadi Assi, Moshe Bechor, Elizabeth Temkin, Alona Keren-Paz, Mitchell J Schwaber, Yehuda Carmeli, the Israel PACH CRAB Working Group, PACH working group, Jacob Haviv, Esther-Lee Marcus, Hana Yosef, Ari Lauden, Debby Ben-David, Angela Shimonov, Jochanan Stessman, Esther Ben Hamo, Moria Atun,, Maya Shkolnik Gazit, Oshra Tirosh, Yana Vishnevski, Irena Uzlianer, Nadya Kagansky, Yochai Levy, Tanya Bogoslavsky, Orna Eluk, Ayala Gisele Sasson, Riham Matar Matanis, Margalit Ben Shimol, Mohamed Nassar, Tatiana Nagulevich, Osnat Kimchi, Boris Svirsky, Mariana Habiballa, Rabea Ramlawe, Ibrahim Saffuri, Subhi Azzam, Tatiana Hutzistov, Pnina Ciobotaro, Pasha Gur, Ilana Or, Nurit Ben Aroya, Yulia Vipritski, Olga Druker, Inna Kaganovich Zafrany, Inna Shugaev, Eduard Zalyesov, Svetlana Zheleznyak, Alona Paz, Ariel Yakim

**Affiliations:** 1 National Institute for Antibiotic Resistance and Infection Control, Ministry of Health, Tel Aviv, Israel; 2 Faculty of Medical and Health Sciences, Tel Aviv University, Tel Aviv, Israel; 3 The members of the working group are listed under Collaborators

**Keywords:** carbapenem-resistant *Acinetobacter baumannii*, post-acute care hospitals, antibiotic resistance, point prevalence survey, infection control

## Abstract

**Background:**

Post-acute care hospitals (PACH) may act as regional reservoirs for multidrug-resistant organisms.

**Aim:**

We aimed to investigate the prevalence of carbapenem-resistant *Acinetobacter baumannii* (CRAB) carriers and identify risk factors for CRAB carriage in PACH.

**Methods:**

We conducted a point prevalence survey in 18 PACH in Israel from June to December 2021. We screened patients in 55 wards of four types (mechanical ventilation, skilled nursing, sub-acute and rehabilitation) for CRAB carriage from skin, rectum and tracheostomy secretions (if applicable). We collected data on patient characteristics (including prior CRAB carriage) and ward and institution characteristics. We calculated the prevalence of CRAB carriers, the percentage of newly detected carriers, and assessed predictors of CRAB carriage using a mixed-effects logistic regression model.

**Results:**

We screened 1,725 patients, with 385 (22%) testing positive for CRAB. The median prevalence of CRAB carriers was 48% (interquartile range (IQR): 33–70) in ventilation wards, 28% (IQR: 18–46) in skilled nursing wards, 8% (IQR: 6–13) in sub-acute wards and 0% (IQR: 0–3) in rehabilitation wards. Only 31% (118/385) had a known history of CRAB carriage. Individual risk factors for CRAB positivity included known CRAB carriage, bedsores and presence of a feeding tube. Modifiable ward-level risk factors included poor availability of alcohol-based hand rub (adjusted odds ratio (aOR) = 3.22; 95% confidence interval (CI): 1.52–6.81) and suctioning in common areas (aOR = 2.23; 95% CI: 1.30–3.85).

**Conclusions:**

The hidden reservoir of CRAB carriers in Israeli ventilation and skilled nursing wards is large. We identified modifiable risk factors at ward level, highlighting areas for targeted intervention.

**Key public health message**

**What did you want to address in this study and why?**

Carbapenem-resistant *Acinetobacter baumannii* (CRAB) is a multidrug-resistant organism that causes severe infections in hospitalised and critically ill patients. Although CRAB has been recognised as a problem in post-acute care hospitals (PACH), we still don’t fully understand how common it is among patients. We wanted to find out how widespread CRAB carriers are in PACH and to identify risk factors—especially conditions that can be changed or improved.

**What have we learnt from this study?**

Nearly one-third of patients in skilled nursing wards and almost half of those in prolonged mechanical ventilation wards in Israel tested positive for CRAB carriage. Most carriers were only discovered through our research, so the staff did not use special infection control measures. We found several modifiable factors that were linked to carriage, e.g. the lack of enough alcohol-based hand sanitisers and the practice of performing suctioning in common areas.

**What are the implications of your findings for public health?**

The large number of hidden CRAB carriers in PACH makes it likely that the bacteria will spread. The risk factors we identified can help healthcare workers recognise high-risk PACH patients when they are transferred to acute care hospitals. Policymakers should allocate more resources to high-risk PACH wards to improve early detection of CRAB carriers through screening upon admission and to strengthen infection control measures.

## Introduction

The spread of carbapenem-resistant *Acinetobacter baumannii* (CRAB) is a risk for hospitalised patients, particularly those who are critically ill [1]. These multidrug-resistant bacteria cause severe infections, often with limited treatment options, leading to high case fatality [2,3]. Moreover, they may rapidly disseminate within healthcare facilities during outbreaks and persist for prolonged periods [4-6]. The transfer of CRAB carriers back and forth between acute care hospitals (ACH) and long-term care facilities (LTCF) presents a risk for dissemination of CRAB in both settings [7,8]. This risk is especially notable in post-acute care hospitals (PACH) providing care for patients who require skilled nursing or prolonged mechanical ventilation. In a recent survey conducted in the United States (US), the prevalence of CRAB carriage among ventilated patients was higher in LTCF than in ACH [9]. In some European and Mediterranean countries, CRAB is a well-recognised problem, yet the topic of CRAB in LTCF, particularly in PACH, in these regions has only been minimally explored [10-12].

Knowing the prevalence and risk factors for CRAB colonisation in PACH is important for several reasons: it enables targeted screening and pre-emptive isolation measures of high-risk individuals upon admission to ACH, it supports interventions aimed at reducing carriage rates within PACH, and it assists clinicians in prescribing appropriate empiric therapy for acute infections [13,14]. Risk factors for colonisation or infection with multidrug-resistant organisms (MDRO) may encompass both individual and institutional characteristics. Previous studies primarily focused on individual risk factors, with limited data specifically addressing CRAB [15].

In this study, our primary aim was to investigate the prevalence and predictors of CRAB carriage in PACH by examining institutional, departmental and individual risk factors. Our secondary aim was to compare the prevalence of CRAB carriage based on a point prevalence study with the prevalence based on known carriage history.

## Methods

### Setting and study design

We conducted a 1-day point prevalence survey in all 18 PACH in Israel from June to December 2021. As described in Ben-David et al., PACH in Israel provide intensive and often prolonged care to high-acuity patients after hospital discharge [16]. The survey was part of the initiatives led by the National Center for Infection Control (NCIC) in the Ministry of Health to monitor and control MDRO. The NCIC provided supplies, logistics and microbiological laboratory testing for the survey, and collected relevant data at the patient, ward and institution level.

### Sample

The population comprised patients hospitalised in PACH in the four high acuity ward types: prolonged mechanical ventilation, skilled nursing (e.g. patients with pressure sores, intravenous treatment, or requiring respiratory monitoring), sub-acute care (i.e. patients after an acute illness who require a limited period of nursing care and monitoring) and rehabilitation. For our sample, one of each available ward type per PACH was randomly selected, and all patients within the selected ward were screened for CRAB. In one PACH which only had rehabilitation wards, we sampled three of them.

### Carbapenem-resistant *Acinetobacter baumannii* screening and microbiological methods

Samples for CRAB were collected from the skin using pre-moistened sterile sponges and from the rectum using swabs. Patients with tracheostomy underwent additional sampling of tracheal aspirate. All samples were sent to the national reference laboratory and processed on the day of collection. For details of sampling and microbiological methods see Nutman et al. [17].

### Collection of patient-level data

Patient characteristics were retrieved from medical records using a standardised form. Data collected included age, sex, length of stay (days from ward admission to PPS), presence of bedsores upon admission, date of last hospital discharge, selected comorbidities, functional status (ranging from completely independent to dependent in activities of daily living), presence of nasogastric tube (NGT) or percutaneous endoscopic gastrostomy (PEG), presence of tracheostomy, oral feeding (i.e. eating at least some foods orally), use of H2-receptor antagonist (H2RA) or proton pump inhibitors (PPI), and antibiotic treatment in the last 3 months. History of CRAB carriage and history of other MDRO carriage, including carbapenemase-producing Enterobacterales (CPE), vancomycin-resistant enterococci (VRE), and meticillin-resistant *Staphylococcus aureus* (MRSA), were determined by reviewing both the medical record and surveillance data available at the NCIC.

### Collection of ward-level and institution-level data

Ward-level data were gathered by the NCIC nurse in interviews with the head nurse of the department and by observation during the survey. These data included: ward type, patient density (number of patients/number of rooms), shared bathroom (i.e. bathroom shared by patients from more than one room), hand hygiene facilities (including availability of alcohol based hand-rub (ABHR) in each patient station, antiseptic soap and dedicated sink in each room), protocols for detecting and isolating CRAB carriers, performance of endotracheal suction in common areas (such as the lobby), use of antiseptic soap for patient bathing, and environmental cleaning practices. In addition, data on ward-level antibiotic use, measured as defined daily doses (DDD) per 100 patient-days, were obtained from the NCIC's 2020 annual antibiotic consumption surveillance report [18]. We did not include a measure of staffing levels because these are standardised across institutions by Ministry of Health regulations.

Institution-level data were collected by the NCIC nurse through interviews with the PACH's infection control (IC) nurse or head nurse. These data included ownership (public or private), number of beds, number of high acuity beds, IC organisational structure and staffing, the use of bleach or quaternary ammonium for environmental cleaning, and the performance of hand hygiene and cleaning audits.

### Outcome

The outcome of interest was a positive screening test for CRAB from any body site during the point prevalence survey.

### Statistical analysis

We calculated the sensitivity of CRAB screening at each anatomical site as the number of patients positive at that site divided by the number of patients tested at that site and positive at any site. We performed analysis of variance (ANOVA) to assess differences in the mean percentage of CRAB-positive cases (in all PACH) between different ward types. Subsequently, to discern specific differences between ward types, we performed post-hoc pairwise t-tests with Bonferroni-adjusted p values.

We summarised continuous variables using the mean and standard deviation (SD), and categorical variables by proportions. To identify independent predictors of CRAB carriage, we constructed a mixed-effects logistic regression model, with institution as a random effect. Patient-, ward- and institution-level characteristics were tested in bivariate analysis. Variables with a p value < 0.1 were included in a multivariable analysis. Backwards selection using the Akaike information criterion (AIC) as the selection criterion was employed to refine the final model. We calculated the variance partition coefficient (VPC) to estimate the percentage of variation explained by the random effect (institution) in two different regression models: one including only patient variables, and the other including both patient and ward variables. All analyses were conducted using the R programming language, version 4.3.1.

## Results

A total of 1,725 patients from 55 wards were included in the sample: 409 patients from 15 mechanical ventilation wards, 535 patients from 16 skilled nursing wards, 142 patients from five sub-acute wards, and 639 patients from 19 rehabilitation wards. In the point prevalence survey, 385 (22.3%) patients tested positive for CRAB carriage. The sensitivities of the screening sites were as follows: 95.6% (368/385) for skin, 18.3% (69/377) for the rectum and 33.3% (77/231) for tracheal aspirates.

### Prevalence of carbapenem-resistant *Acinetobacter baumannii* carriage by ward type

The box plot (Figure) depicts the distribution of CRAB prevalence across different ward types. The prevalence was highest in mechanical ventilation wards (median 48%; interquartile range (IQR): 33–70), followed by skilled nursing wards (median: 28%; IQR: 18–46) and sub-acute wards (median: 8%; IQR: 6–13). In rehabilitation wards, CRAB carriage was rare (median: 0%; IQR: 0–3). The prevalence of CRAB carriage was significantly different between mechanical ventilation and sub-acute wards (p = 0.003), mechanical ventilation and rehabilitation wards (p < 0.001), and skilled nursing and rehabilitation wards (p < 0.001).

**Figure:**
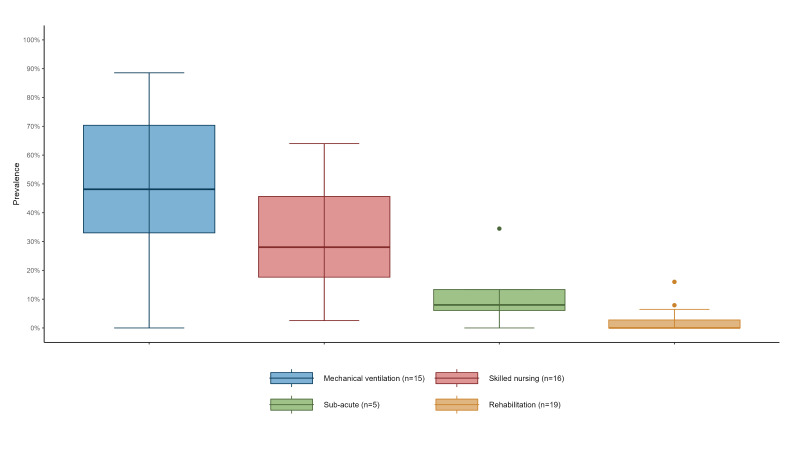
Carbapenem-resistant *Acinetobacter baumannii* prevalence in post-acute care hospitals, by ward type, Israel, 2021 (n = 1,725)

### Previous history of carbapenem-resistant *Acinetobacter baumannii* carriage

Overall, 267 (69.4%) of the 385 patients identified as CRAB carriers in the point prevalence survey had no known history of CRAB carriage. This percentage differed by ward type: 60% (116/195) in mechanical ventilation wards, 79% (126/159) in skilled nursing wards, 78% (14/18) in sub-acute wards, and 85% (11/13) in rehabilitation wards.

### Predictors of carbapenem-resistant *Acinetobacter baumannii* carriage

Table 1, 2 and 3 summarise the bivariate association between individual-, ward- and institution-level characteristics and a positive CRAB screening test. The policy of CRAB screening upon admission was not included as a predictor variable because only one institution implemented this policy.

**Table 1:** Unadjusted association between patient-level characteristics and carbapenem-resistant *Acinetobacter baumannii* carriage, Israel, 2021 (n = 1,725)

Variables^a^		CRAB-positive (n = 385)		CRAB-negative (n = 1,340)		OR (95% CI)	p value
		n	%	n	%		
Demographic characteristics							
Mean age in years (SD)		70.3 (15.7)		73.9 (14.3)		0.97 (0.97–0.98)	< 0.001
Sex	Male	209	54.3	596	44.5	Reference	
	Female	176	45.7	744	55.5	0.67 (0.54–0.85)	0.001
Healthcare characteristics							
Mean length of stay in days (SD)		232.9 (179.7)		147.6 (174.3)		1.003 (1.002–1.003)	< 0.001
Admission from an acute care hospital		329	85.5	1,158	86.4	0.85 (0.59–1.22)	0.383
Hospitalisation in the last year		275	71.4	1,050	78.4	0.60 (0.45–0.79)	< 0.001
Underlying conditions							
Cardiovascular disease		225	58.4	837	62.5	0.87 (0.68–1.12)	0.288
Chronic lung disease		121	31.4	308	23.0	1.40 (1.07–1.82)	0.014
Connective tissue disease		63	16.4	166	12.4	1.60 (1.03–2.47)	0.035
Dementia		82	21.3	212	15.8	1.30 (0.95–1.78)	0.102
Diabetes mellitus		162	42.1	653	48.7	0.81 (0.63,1.03)	0.081
Renal failure		94	24.4	325	24.3	0.92 (0.69–1.21)	0.544
Chronic liver disease		13	3.4	50	3.7	0.85 (0.44–1.63)	0.626
Solid malignancy		48	12.5	192	14.3	0.86 (0.60–1.23)	0.415
Haematologic malignancy		9	2.3	28	2.1	1.23 (0.55–2.76)	0.615
Bedsore upon admission		57	14.8	104	7.8	2.21 (1.51–3.23)	< 0.001
Fully dependent in activities of daily living		327	84.9	772	57.6	5.13 (3.58–7.35)	< 0.001
Treatments and devices							
NGT or PEG		250	64.9	295	22.0	8.73 (6.59–11.57)	< 0.001
Tracheostomy		239	62.1	277	20.7	8.68 (6.51–11.57)	< 0.001
PPI or H_2_RA treatment		190	49.4	715	53.4	0.94 (0.73 1.20)	0.614
Oral feeding		154	40.0	1,061	79.2	0.18 (0.14–0.22)	< 0.001
MDRO history							
History of CRAB carriage		118	30.6	94	7.0	5.74 (4.12–8.00)	< 0.001
History of other MDRO carriage (CPE, VRE, MRSA)		164	42.6	278	20.7	2.81 (2.17–3.63)	< 0.001
Antibiotic history							
Any antibiotic (last 3 months)		111	28.8	240	17.9	1.73 (1.35–2.23)	< 0.001
Aminoglycosides		48	12.5	59	4.4	3.09 (1.99–4.80)	< 0.001
Beta lactamase inhibitors		62	16.1	114	8.5	2.17 (1.51–3.12)	< 0.001
Cephalosporins		72	18.7	221	16.5	1.18 (0.87–1.62)	0.290
Chloramphenicol		10	2.6	10	0.7	4.00 (1.56–10.22)	0.004
Quinolones		44	11.4	113	8.4	1.33 (0.90–1.97)	0.157
Carbapenems		8	2.1	11	0.8	2.78 (1.06–7.28)	0.038
Metronidazole		13	3.4	36	2.7	1.14 (0.58–2.25)	0.707
Glycopeptides		15	3.9	24	1.8	2.18 (1.09–4.34)	0.028
Other antibiotic		15	3.9	63	4.7	0.93 (0.51–1.71)	0.825

CI: confidence interval; CPE: carbapenemase-producing Enterobacterales; CRAB: carbapenem-resistant Acinetobacter baumannii; H_2_RA: H2-receptor antagonist; MDRO: multidrug-resistant organisms; MRSA: meticillin-resistant *Staphylococcus aureus*; NGT: nasogastric tube; OR: odds ratio; PEG: percutaneous endoscopic gastrostomy; PPI: proton pump inhibitors; SD: standard deviation; VRE: vancomycin-resistant enterococci.

^a^ Categorical variables shown as n (%) and continuous variables shown as mean (SD).

Institution was included as a random effect.

**Table 2:** Unadjusted association between ward-level characteristics and carbapenem-resistant *Acinetobacter baumannii* carriage, Israel, 2021 (n = 1,725)

Variables^a^		CRAB positive (n = 385)		CRAB negative (n = 1,340)		OR (95% CI)	p value
		n	%	n	%		
Ward characteristics							
Ward type	Rehabilitation	13	3.4	626	46.7	Reference	
	Sub-acute	18	4.7	124	9.3	7.47 (3.38–16.49)	< 0.001
	Skilled nursing	159	41.3	376	28.1	22.82 (12.51–41.63)	< 0.001
	Mechanical ventilation	195	50.6	214	16.0	53.53 (29.16–98.27)	< 0.001
Mean density (patients per room) (SD)		2.4 (0.6)		2.3 (0.6)		1.24 (0.94–1.62)	0.125
Shared bathroom		197	51.2	471	35.1	5.09 (3.49–7.42)	< 0.001
Mean percentage of known CRAB carriers (SD)		25.2 (20.0)		8.6 (13.4)		1.07 (1.06–1.08)	< 0.001
Mean percentage of carriers of other MDRO (SD)		38.3 (15.2)		22.1 (13.6)		1.08 (1.07–1.10)	< 0.001
Mean percentage of patients with tracheostomy		58.3 (41.8)		21.8 (35.3)		1.03 (1.02–1.03)	< 0.001
Mean antibiotic use - DDD/100 patient days (SD)		12.5 (6.7)		13.5 (8.2)		0.98 (0.95–1.00)	0.036
Infection control measures							
Isolation of CRAB carriers in single patient rooms or cohort	Yes or partial	83	21.6	288	21.5	Reference	
	No	302	78.4	1,052	78.5	0.68 (0.47–0.97)	0.033
No ABHR available at each patient station		125	32.5	196	14.6	4.38 (2.10–9.13)	< 0.001
Dedicated sink for handwashing in patient rooms	All rooms	276	71.7	841	62.8	Reference	
	Some rooms	35	9.1	46	3.4	9.29 (4.37–19.75)	< 0.001
	None	74	19.2	453	33.8	0.68 (0.43–1.08)	0.101
No antiseptic soap for hand hygiene		68	17.7	145	10.8	1.04 (0.71–1.52)	0.852
Performance of suction in common areas		218	56.6	392	29.3	3.93 (2.78–5.54)	< 0.001
Antiseptic wash for bathing	All patients	10	2.6	44	3.3	Reference	
	MDRO carriers	146	37.9	244	18.2	0.36 (0.08–1.62)	0.181
	None	229	59.5	1,052	78.5	0.09 (0.02–0.43)	0.003
Environmental cleaning							
Hoist slings shared between patients		85	22.1	199	14.9	1.10 (0.72–1.69)	0.649
Routine cleaning of patient station – not every day		22	5.7	140	10.4	0.34 (0.13–0.91)	0.032
Routine cleaning of medical equipment between patients - no		67	17.4	169	12.6	3.90 (2.15–7.08)	< 0.001
Curtain change	On discharge	178	46.2	495	36.9	Reference	
	On discharge of MDRO carrier	184	47.8	649	48.4	0.75 (0.52–1.08)	0.118
	No clear policy	23	6.0	196	14.6	0.05 (0.02–0.13)	< 0.001

ABHR: alcohol-based hand-rub; CI: confidence interval; CRAB: carbapenem-resistant Acinetobacter baumannii; DDD: defined daily dose; MDRO: multidrug-resistant organisms; OR: odds ratio; SD: standard deviation.

^a^ Categorical variables shown as n (%) and continuous variables shown as mean (SD).

Institution was included as a random effect.

**Table 3:** Unadjusted association between institution-level characteristics and carbapenem-resistant *Acinetobacter baumannii* carriage, Israel, 2021 (n = 1,725)

Variables^a^		CRAB positive (n = 385)		CRAB negative (n = 1,340)		OR (95% CI)	p value
		n	%	n	%		
Mean number of beds (SD)		276.0 (87.1)		289.0 (112.5)		1.00 (0.99–1.00)	0.514
Mean percentage of high acuity beds (SD)		0.8 (0.2)		0.8 (0.2)		1.10 (0.13,9.30)	0.929
Mean IC nurses per 100 high acuity beds (SD)		0.30 (0.25)		0.33 (0.26)		0.36 (0.06–2.25)	0.276
Presence of a mechanical ventilation ward		366	95.1	1,139	85.0	3.41 (1.00–11.63)	0.050
Private ownership		239	62.1	727	54.3	1.76 (0.70–4.46)	0.231
Lack of IC organisational infrastructure		210	54.5	584	43.6	1.85 (0.77–4.48)	0.171
Environmental disinfection	Bleach	276	71.7	1,153	86.0	Reference	
	Quaternary ammonium	109	28.3	187	14.0	2.93 (0.99–8.67)	0.052
Hand hygiene observations - no		220	57.1	653	48.7	1.68 (0.68–4.17)	0.261
Cleaning audits - no		245	63.6	856	63.9	1.25 (0.47–3.35)	0.658

CI: confidence interval; CRAB: carbapenem-resistant Acinetobacter baumannii; IC: infection control; OR: odds ratio; SD: standard deviation.

^a^ Categorical variables shown as n (%) and continuous variables shown as mean (SD).

Institution was included as a random effect.

In the multivariable regression analysis, only individual and ward level characteristics were significantly associated with CRAB carriage (Table 4). Among patient-level characteristics, female sex and extended length of stay were protective, whereas the presence of bedsores, NGT or PEG, and known carriage of CRAB were risk factors. Ward-level characteristics associated with CRAB carriage included ward type (sub-acute care, skilled nursing and mechanical ventilation), percentage of known MDRO carriers, absence of ABHR at each patient station, and the performance of suction in common areas. The pseudo-R^2^, indicating the proportion of CRAB carriage explained by the model, was fair (0.59). The VPC of the regression model, which indicates the percentage of variation explained by the random effect (institution), was 27.1% if only patient-level variables were included. Adding ward-level variables reduced the VPC to 11.5%, underscoring the impact of ward-level differences on the prevalence of CRAB in PACH.

**Table 4:** Multivariable regression model of factors associated with carbapenem-resistant *Acinetobacter baumannii* carriage, Israel, 2021 (n = 1,725)

Variables		aOR (95% CI)	p value
Individual level characteristics			
Sex	Male	Reference	
	Female	0.52 (0.39–0.71)	< 0.001
Length of stay		0.999 (0.998–1.000)	0.031
Bedsore upon admission		2.14 (1.35–3.39)	0.001
NGT or PEG		2.63 (1.77–3.90)	< 0.001
Tracheostomy		1.68 (0.96–2.95)	0.069
History of CRAB carriage		1.93 (1.30–2.87)	0.001
History of other MDRO carriage (CPE, VRE, MRSA)		1.29 (0.94–1.78)	0.121
Beta-lactamase inhibitors (last 3 months)		1.41 (0.91–2.19)	0.125
Ward level characteristics			
Ward type	Rehabilitation	Reference	
	Sub-acute	4.78 (2.03–11.21)	< 0.001
	Skilled nursing	6.88 (3.35–14.10)	< 0.001
	Mechanical ventilation	4.46 (1.81–11.00)	0.001
Mean percentage of carriers of other MDRO		1.04 (1.02–1.05)	< 0.001
No ABHR available at each patient station		3.20 (1.52–6.76)	0.002
Dedicated sink for handwashing in patient rooms	All rooms	Reference	
	Some rooms	2.19 (0.92–5.20)	0.076
	None	0.88 (0.48–1.61)	0.676
Performance of suction in common areas		2.25 (1.31–3.87)	0.003

ABHR: alcohol-based hand-rub; aOR: adjusted odds ratio; CI: confidence interval; CPE: carbapenemase-producing Enterobacterales; CRAB: carbapenem-resistant Acinetobacter baumannii; MDRO: multidrug-resistant organisms; MRSA: meticillin-resistant *Staphylococcus aureus*; NGT: nasogastric tube; PEG: percutaneous endoscopic gastrostomy; VRE: vancomycin-resistant enterococci.

Institution was included as a random effect.

## Discussion

In this national survey of all PACH in Israel, we found a high prevalence of CRAB carriage in mechanical ventilation and skilled nursing wards. The median prevalence of CRAB carriage reached nearly 50% in mechanical ventilation wards and 30% in skilled nursing wards. Notably, most CRAB carriers had gone undetected before the survey. Various individual- and ward-related characteristics were associated with CRAB carriage. Modifiable ward-level risk factors included the poor availability of ABHR and the performance of suctioning in common areas.

The emergence of multidrug-resistant *A. baumannii*, including CRAB, in LTCF has been documented for two decades [7,19,20]. However, the full extent of this problem remains unclear. Several US studies of high-risk LTCF patients, including those in skilled nursing and sub-acute care, found CRAB prevalence between 7% and 12% [21,22]. Reports from Europe have shown lower prevalence, such as 3% in LTCF from Italy (in 2016), which may partly reflect differences in detection methods [12]. Two statewide surveys of ventilated patients in Maryland found CRAB prevalence rates in LTCF of 43% in 2010 and 35% in 2023 [9,23]. Our study confirmed that CRAB prevalence is very high among ventilated patients in PACH, but it is not limited to this group.

The hidden reservoir of carriers of MDRO in LTCF is a major problem. In a large survey of MDRO prevalence (including MRSA, VRE, extended-spectrum β-lactamase–producing organisms, and carbapenem-resistant Enterobacterales (CRE), but not CRAB) conducted in 21 LTCF in California, 83% of cases of MDRO carriage were previously unknown to the facility. Moreover, 66% of colonised patients had no prior history of any MDRO colonisation, indicating that appropriate contact precautions were not enforced [24]. Our study demonstrated that CRAB under-detection is also a problem in PACH across all high acuity ward types, highlighting an area for intervention. We have previously shown that early detection of carriers, combined with contact precautions tailored to the type of patients, are effective strategies to combat the spread of CRE in LTCF in Israel [16]. Interventions for active surveillance of CRAB have proven successful in ACH [25,26]. In our study, we could not evaluate the effect of active surveillance on CRAB prevalence because only one PACH had a policy of screening upon admission. Further studies are necessary to evaluate the effectiveness of CRAB active surveillance in LTCF, particularly in PACH. Nevertheless, it seems reasonable to recommend universal CRAB screening upon admission to ventilated wards, where CRAB prevalence was highest.

A review by van Buul et al. summarised the risk factors associated with colonisation or infection with any MDRO in LTCF [15]. Commonly identified individual risk factors were prior antibiotic use, the presence of invasive devices, lower functional status, the presence of decubitus ulcers or wounds, prior hospitalisation, and prior colonisation with MDRO. Looking specifically at risk factors for CRAB carriage, data are limited and include small studies [21,22]. In these two studies, co-colonisation with CRAB and other MDRO was common. Functional disability and ventilation support were among the few identified risk factors.

Few studies have addressed institutional or ward-level risk factors for MDRO carriage in LTCF. Loeb et al. examined the effect of three variables – staffing, hand washing and antimicrobial exposure – on MDRO carriage [27]. They found that having more nurses and using antimicrobial soap reduced the risk of MRSA, and that more hand-washing sinks reduced the risk of trimethoprim/sulfamethoxazole-resistant Enterobacterales. Von Baum et al. looked at risk factors for MRSA carriage, including nursing home size and multiple individual characteristics [28]. They found that risk was higher in nursing homes with 41–100 beds than in institutions with more than 100 beds.

In our study, we examined multiple variables at different levels: individual, ward and institution. We identified the following individual risk factors: bedsores, having an NGT or a PEG, and a history of CRAB carriage. A prolonged stay was a risk factor for CRAB carriage in bivariate analysis, and a protective factor in multivariate analysis, but the OR in both was close to 1.0. The minimal influence of length of stay may reflect the dynamic nature of MDRO carriage in PACH. A study of patients in US nursing facilities found a high prevalence of MDRO upon admission, and both clearance of carriage and new acquisitions during their stay [29].

Several ward-level characteristics were significantly associated with CRAB carriage: ward type, mean percentage of carriers of other MDRO (but not CRAB) in the same ward, lack of ABHR at the bedside and suctioning in common areas. Lack of ABHR may be a proxy for low hand hygiene compliance [30]. Common area suctioning may increase risk by contaminating the shared environment, such as the lobby [31]. These two modifiable risk factors are targets for intervention. Overall, adding ward-level variables to the regression model reduced the percentage of variance explained by the random effect by half (from 27.1% to 11.5%). This suggests that differences between wards account for a substantial amount of the variation in the prevalence of CRAB.

This study has several strengths. Firstly, it has a national scope, encompassing all PACH in Israel and four types of high acuity wards. Secondly, the use of sensitive sampling and diagnostic methods ensured accurate detection of CRAB carriage [17]. Finally, we examined variables at three levels.

There are also several weaknesses. Firstly, a point prevalence study cannot answer whether residents acquired CRAB before or during their PACH stay. Secondly, only one PACH performed routine CRAB screening upon admission; therefore, we defined patients as not known carriers if they were positive for CRAB upon admission but not screened and did not have a recorded history of CRAB. This reflects the actual information available to the PACH. Thirdly, data on recorded history of CRAB may have been incomplete. To mitigate this possibility, we relied not only on patient records but also on the national surveillance system of CRAB reported by ACH. Fourthly, documentation in the medical chart of other patient characteristics, such as co-morbidities and antibiotic exposure, may also have been incomplete. Finally, no institution-level risk factors remained significant in multivariable analysis; there may be other institution characteristics that affect CRAB prevalence that we did not measure, such as hand hygiene compliance.

Our findings have important implications for IC practitioners, clinicians and policymakers. The risk factors that we found can help IC practitioners identify high-risk PACH patients upon transfer to ACH, which can perform screening and consider pre-emptive isolation. Clinicians can utilise our findings to help ensure appropriate empiric antibiotic treatment for high-risk patients exhibiting clinical signs of infection. Policymakers should invest resources in high-risk PACH wards to improve early detection and enhance infection control practices in these settings. Since this PPS in 2021, the NCIC has implemented a national intervention to control CRAB, including in PACH. The PACH report monthly incident and prevalent CRAB cases. The NCIC notifies PACH when their transferred patients screened positive for CRAB upon admission to an ACH. The PACH have implemented various measures such as wide distribution of ABHR, active surveillance for CRAB, and cohorting of CRAB carriers (when feasible). We are planning a second PPS in 2025 to assess the impact of these interventions.

An area for future research is serial screening of PACH patients for CRAB upon admission and periodically thereafter to understand the dynamics of CRAB acquisition and transmission. These studies should include molecular analysis of CRAB strains to confirm cross-transmission. Cluster randomised trials are needed to evaluate the effectiveness of various infection control interventions in PACH, such as active surveillance, environmental decontamination, and changes in suction practices in reducing CRAB prevalence.

## Conclusion

The large hidden reservoir of CRAB in PACH makes transmission within the institution and between PACH and ACH nearly inevitable. The risk factors we identified highlight high-risk wards within PACH, where enhanced screening and infection control measures are needed to uncover this reservoir and decrease its size. Acute care hospitals can add another layer of protection against CRAB transmission by targeted screening and possibly preemptive isolation of high-risk patients transferred from PACH. 

## PACH working group

Jacob Haviv, Esther-Lee Marcus, Hana Yosef (Herzog Medical Center); Ari Lauden (Reut Rehabilitation Hospital); Debby Ben-David, Angela Shimonov (Beit Rivka Geriatric Medical Center); Jochanan Stessman, Esther Ben Hamo, Moria Atun (Hod Amal Ma'ale Adumim Geriatric Hospital); Maya Shkolnik Gazit, Oshra Tirosh, Yana Vishnevski, Irena Uzlianer (Beit Hadar Medical Rehabilitation and Nursing Center); Nadya Kagansky, Yochai Levy (Shmuel Harofe Geriatric Medical Center); Tanya Bogoslavsky, Orna Eluk (Shoham Geriatric Medical Center); Ayala Gisele Sasson (Bayit Balev Rishon Le’zion Geriatric Rehabilitation Center); Riham Matar Matanis(Bayit Balev Nesher Geriatric Rehabilitation Center); Margalit Ben Shimol (Dorot Geriatric Rehabilitation Center); Mohamed Nassar, Tatiana Nagulevich, Osnat Kimchi (Golden Care Ness Ziona Geriatric Rehabilitation Center); Boris Svirsky, Mariana Habiballa, Rabea Ramlawe, Ibrahim Saffuri, Subhi Azzam (Golden Care Dushi Nazareth Geriatric Rehabilitation Center); Tatiana Hutzistov (Golden Care Tel Aviv Geriatric Rehabilitation Center); Pnina Ciobotaro, Pasha Gur (Herzfeld Geriatric Rehabilitation Medical Center); Ilana Or, Nurit Ben Aroya (Loewenstein Rehabilitation Hospital); Yulia Vipritski, Olga Druker (Neve Amit Geriatric Center); Inna Kaganovich Zafrany (Neve Shva Geriatric Center); Inna Shugaev, Eduard Zalyesov, Svetlana Zheleznyak, Alona Paz (Fliman Geriatric Rehabilitation Hospital); Ariel Yakim (Geriatrics Branch, Ministry of Health).
